# Equipping Persons with Sickle Cell Disease, Transforming Care: A Protocol Paper on Feasibility and Acceptability of Self-Management Package in the Tribal Communities of Southern India

**DOI:** 10.12688/wellcomeopenres.24369.1

**Published:** 2025-10-27

**Authors:** Manashri Bhuyar, Tanya Seshadri, Pooja Aggarwal, Anandhu KR, Deepa Bhat

**Affiliations:** 1 Centre for Training, research, and Innovation in Tribal Health, Institute of Public Health Bengaluru, Bengaluru, Karnataka, India; 2 Department of Anatomy, JSS Medical College, JSS Academy of Higher Education and Research, Mysuru, Karnataka, India; 3 JSS Hospital, Mysuru, Karnataka, India

**Keywords:** Sickle Cell Disease, Self-management, Acceptability, Feasibility, Implementation Research

## Abstract

**Introduction:**

Sickle cell disease affects tribal communities unequally. Self-management empowers the patient to take corrective actions, improve decision-making, and deepen understanding of the disease. Considering the unique challenges of the tribal community, implementing self-management complementary to the prescribed clinical management for sickle cell disease care can lead to greater autonomy, improved health outcomes, and, ultimately, quality of life. Hence, this study aims to develop a feasible and acceptable self-management package for tribal persons living with Sickle cell disease and caregivers residing in the Mysuru and Chamarajanagar districts of Karnataka.

**Method:**

The study will be conducted in two phases. In the first phase, a self-management package will be developed with insights from health providers working with Persons living with sickle cell disease, non-government organisations, Subject experts and persons with sickle cell disease and caregiver. This package will be implemented in the second phase for persons living with sickle cell disease. Participants will undergo capacity-building training and receive regular follow-up for 12 months through monthly telephonic calls and a few home visits. Assessment will be done with Acceptability Intervention Measurement (AIM) and Feasibility Intervention Measurement (FIM) at regular intervals. Suggestions and feedback will be added to make the package more comprehensive. Thus, the expected outcome is an acceptable and feasible self-management package.

**Conclusion:**

This proposed study is an important step towards addressing a critical gap in sickle cell disease management in tribal populations in India. The anticipated outcome of this research is to enhance self-management practices in individuals with sickle cell disease.

## Plain Language Summary

A genetic condition known as Sickle Cell Disease (SCD) is a genetic condition that can lead to complications such as intense pain, fatigue, and sometimes death. In India, disease prevalence is notably high, ranking second to Sub-Saharan Africa, and it is particularly high among the tribal population. This lifelong condition often results in multiple hospitalisations, leads to out-of-pocket expenditures, affects work capacity, and ultimately diminishes quality of life. The ‘ *National Sickle Cell Anaemia Elimination Mission*’ initiated services focusing on screening for SCD and care to address these challenges.

However, improving individuals' ability to effectively use these services and make informed health decisions is equally important. Research indicates that self-management can be an effective tool in this regard. This approach can empower individuals and their caregivers to manage their condition more effectively and make informed decisions. Building self-management capacity among persons with SCD can ensure better utilisation and engagement by these individuals and their caregivers.

This proposed study aims to design a self-management package for SCD in the tribal population. This package does not intend to interfere with prescribed medical treatment and advice. Furthermore, the proposed study also intends to assess its acceptability and feasibility.

According to the objective, the study will be conducted in two phases. In the first phase, we will design a self-management package by finalising the content, delivery process, and strategies for adhering to those practices. The second phase will assess the acceptability and feasibility of the designed self-management package by providing the necessary training for using the self-management package to all persons living with SCD in the given study setting. Proper measurement tools will be employed to measure acceptability and feasibility, with regular follow-up planned to enhance engagement. Thus, the outcome will be an acceptable and feasible self-management package.

## Background

Sickle Cell Disease (SCD) is a genetic condition linked to morbidity and mortality. The prevalence of SCD has increased from 5.46 million (year 2000) to 7.74 million cases (year 2021) globally, marking a rise of almost 41%. SCD ranked 12th for mortality among children less than five years of age worldwide, across all causes, according to the estimation by Global Burden of Disease in 2021 ^1^. Following Sub-Saharan Africa, India bears the second-highest burden of SCD. The estimated disease prevalence in India is 1.17%, while the trait prevalence is 5.9%. Within the tribal communities, the disease estimation is higher at 4.05%, compared to the 0.84% seen in non-tribal populations. A similar trend is observed with sickle-cell trait prevalence, which is 8.61% among tribal communities and 4.81% among non-tribal communities ^2^. Furthermore, the prevalence of the sickle-cell trait varies significantly among different tribal communities, ranging from 1–35% ^3^.

SCD causes red blood cells (RBC) to become rigid and crescent-shaped, reducing their life span from 120 to 10–20 days ^4, 5^. The impaired flexibility of RBCs leads to a broad spectrum of clinical manifestations, both acute and chronic complications, with pain and fatigue being common symptoms. This variance is influenced by genetic, environmental, and healthcare factors ^6, 7^. While earlier clinical studies suggested that the Indian sickle cell haplotype has milder symptoms than the African haplotype, few studies have challenged this argument and recommended further investigations ^8^. Several treatments, including pain management, hydroxyurea, blood transfusions, and bone marrow transplantation, have shown effectiveness in enhancing the condition ^9^.

In response to the SCD’s burden, the Government of India launched the National Sickle Cell Anaemia Elimination Mission (NSCAEM) to eliminate Sickle Cell Anaemia before 2047. Although the initiative primarily focuses on tribal communities conducting screening, management and counselling ^10^. A significant gap exists between the tribal beneficiaries and health service provision. This is often attributed to geographical isolation, cultural differences, and inadequate healthcare infrastructure ^11, 12^.

This inequity results in delays in medical attention and reduced access to healthcare services, ultimately affecting health outcomes negatively. Moreover, the absence of context-specific practices compounded the challenges. In such circumstances, SCD disproportionately affects the tribal community due to the frequent need to visit healthcare facilities, which disrupts their daily routines, also leading to out-of-pocket expenditure ^13^. To address this gap, a tailored approach to achieve the specific health-related needs of the tribal community is required ^14^. Given the unique challenges faced by the tribal people with SCD, self-management can play a vital role in SCD care and improving health outcomes. It can enable patients to take timely corrective action based on their symptoms, improve their decision-making, and enhance their understanding of the disease ^15, 16^.

Several studies have shown the effectiveness of self-management in SCD ^17– 19^. However, most studies are conducted in the international context, predominantly in hospital settings, and very few are in a community context. Existing self-management models from other countries may not directly apply to the Indian tribal communities due to different dietary habits, economic conditions, and socio-cultural factors. Therefore, context-specific, evidence-based interventions are essential for effective self-management for the tribal communities, especially given the complexities associated with SCD ^20^.

Therefore, the study's objectives are to design a self-management package for tribal PwSCD and assess its acceptability and feasibility among PwSCD and caregivers in tribal communities. The package will be iteratively refined to enhance its applicability and relevance.

The study adopts an operational definition of self-management that complements clinical management but no where replaces it, akin to that proposed by Poku et al., stating, “ *Self-management is the purposeful performance of specific learned tasks, activities, and behaviours to manage the medical, psychological, and life impacts of chronic illness*.” ^15^

In some countries in North and South America, there is consensus on using the term “Indigenous” to refer to the region's original inhabitants. However, in the context of India, the term “tribal” is commonly used to describe communities classified under the “Scheduled Tribes” category in the Constitution of India due to the complexity of migration history ^21, 22^. In this manuscript, we have chosen to use the term “Tribal,” as it is widely used in academic, legal, and official documents within India.

## Methods

**Figure 1.**
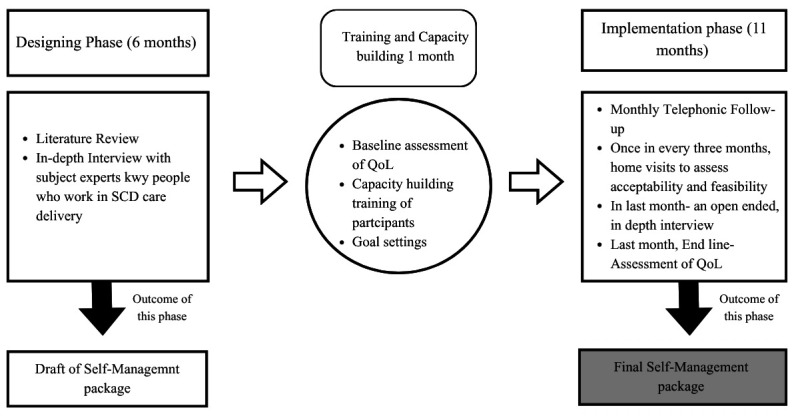
Schematic flow of study phases. The study will start with the designing phase (6 months), followed by 1 month of capacity building of participants. The drafted self-management package will be implemented in the subsequent phase of 11 months for one participant. Specific activities for each phase have been outlined. A final self-management package is an expected outcome of the study. QoL, Quality of Life; SCD, Sickle Cell Disease. The figure was created using the ‘pro’ version of the Software Canva.

This study will be carried out in two phases: The design phase (Phase I) and the self-management package refinement phase, which includes implementation (Phase II). Phase I will focus on designing a self-management package by gathering qualitative insights and a literature review. Phase II will employ quantitative methods to assess acceptability and feasibility in a real-world setting, supplemented by open-ended questions to make it more acceptable and feasible ( Figure 1).

**Figure 2.**
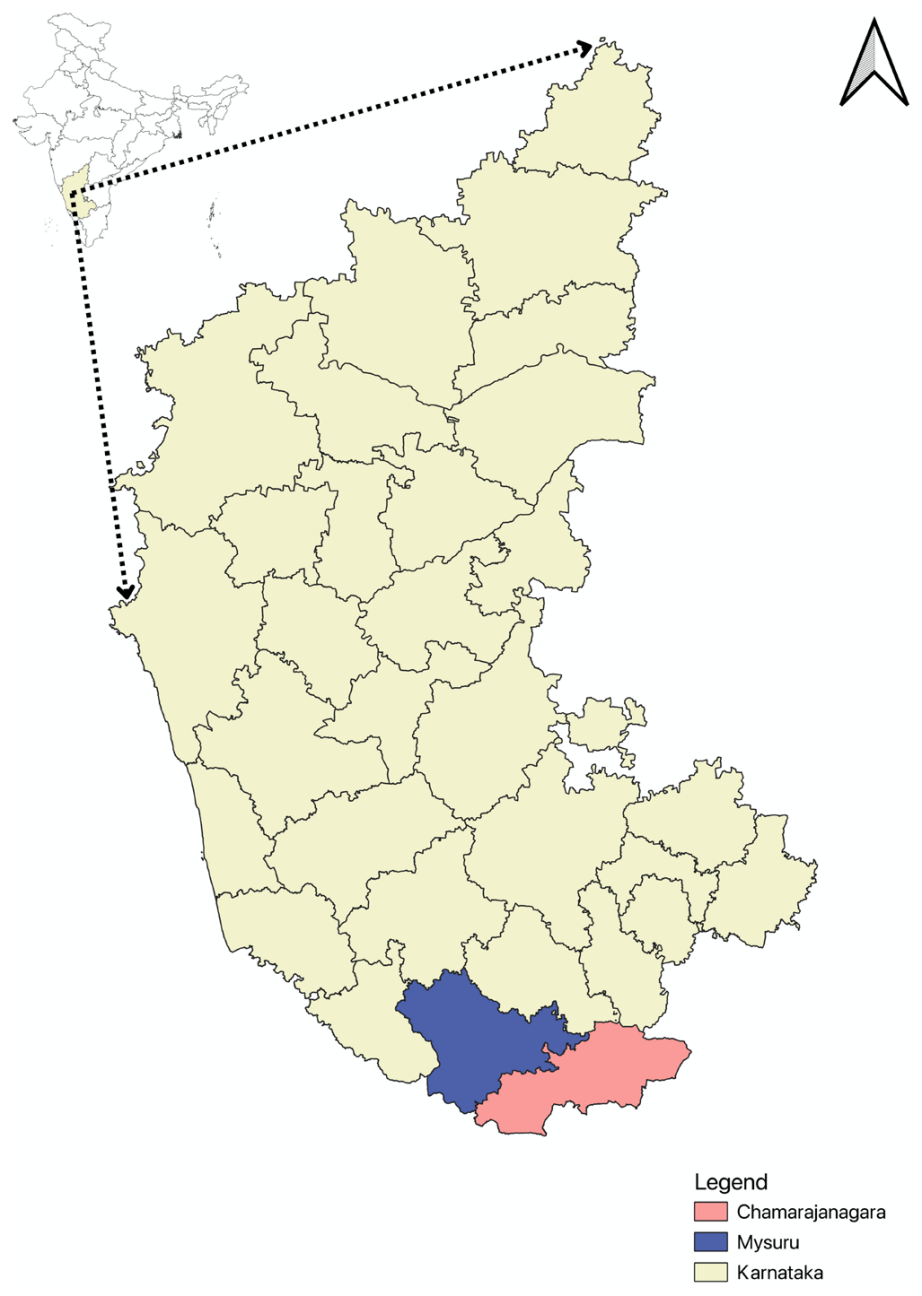
Map of study location. The study will be conducted in two southernmost districts of Karnataka- Chamarajanagar and Mysuru (shown in the inset). The Scheduled tribes populations in Chamarajanagar and Mysuru are 11.78% and 11.15% respectively ^23, 24^. The map showing the two study sites was created using QGIS ( https://qgis.org/). India-based geography layer obtained from geoBoundaries ( https://www.geoboundaries.org/) ^25^; Karnataka state and district layers retrieved from KGIS ( https://kgis.ksrsac.in/kgis/downloads.aspx).

**Study settings**: We intend to prepare the self-management package for PwSCD and caregivers of tribal communities residing mainly in blocks of Chamarajanagar and Mysuru district of Karnataka state, India. Chamarajanagar district shares a border with Kerala and Tamil Nadu. Most of the area of both districts is hilly and forested. Communities such as the Soliga, Jen Kruba, and Betta Kuruba are in these districts,are subgrouped in the list “Scheduled tribes” in the Indian Constitution. Their livelihood is mostly forest-dependent. Even though we will gather inputs for other key personnel working on Sickle cell disease care delivery of area outside the study setting, inputs from local PwSCD and caregivers will be collected through In-depth- interview, to design a self-management package and then this package will be tested for tribal communities of selected blocks of these districts ( Figure 2).

To select the blocks of the districts, purposive and convenient sampling techniques will be used. It will depend on the rapport with the PwSCD and caregivers and the optimal availability of intended participants.

### Phase I: Designing phase

Phase I aims to design a self-management package for PwSCD in tribal communities. Two methods will be used in the designing phase: 1. a review of literature, and 2. in-depth interviews/focus group discussions.

*Review of Literature:* A literature search will identify the key topics and strategies to implement that should be included in the Self-management package and explore implementation strategies. Databases such as PubMed, Google Scholar, Scopus and Web of Science will be searched using relevant keywords, including but not limited to ‘Self-management and Sickle cell disease’ and ‘Self-management and non-communicable disease’. The review will include original articles, policy documents, opinions, and grey literature. Additionally, websites of international health departments and NGOs working in SCD care delivery. All sources will be managed using Zotero software, and studies will be screened based on title and abstract for inclusion criteria and relevance to the study objectives.

*In-depth interviews(IDIs):* IDIs will be conducted with subject experts and key resource persons, including representatives from local non-governmental organisations (NGOs) involved in delivering SCD care across India to prioritise self-management practices, their components and delivery mechanisms from the local tribal context. Additionally, the IDIs will be conducted with persons with Sickle cell Disease (PwSCD)and their caregivers living in the districts of Chamarajanagar and Mysuru. Participants will be purposively selected to ensure diversity in sociodemographic characteristics, such as age, educational status, and employment status. Their interviews aim to capture the lived experiences, challenges, and facilitators of those involved in adopting and adhering to self-management practices. Until the thematic saturation achievement, data collection will continue.

*Focus group discussion (FGD)*: A FGD involving a mixed group of health care providers (both government and non-government) and/or caregivers will be conducted, contingent upon the preliminary findings from the in-depth interviews. The decision to include FGD will be based on its potential to enrich qualitative insights and further elucidate emerging themes relevant to self-management practices in SCD.

For analysis, all qualitative data collected in the local language will be transcribed and translated into English. Thematic analysis will be conducted using Qual Coder Software version 3.6 ^26^. Preliminary themes will guide the development of the initial draft of the self-management package, which will then be reviewed by the subject experts and a few PwSCD and Caregivers for validation and feedback. Based on this consultation, the package will be revised to enhance relevance and usability. Potential domains of self-management may include prevention of complications, symptom monitoring, management of mild symptoms with prescribed medication and context-based decision making. Emerging themes and subthemes will be iteratively incorporated into the design.

At the end of this phase, the research team will decide on the content sequence and strategies of the self-management package and prepare a final design. This designing phase is expected to take 6–7 months, after which the implementation phase will commence, beginning with the training of field assistants.

### Phase II: Implementation phase (12 months)

Phase II aims to assess the acceptability and feasibility of the self-management package developed among PwSCD and caregivers from tribal communities in the study setting. It will be implemented in a block of Mysuru and Chamarajanagar districts of Southern Karnataka in India, specifically in tribal communities. An effort to establish the ‘Population-Based Hemoglobinopathy Registry’ is underway through a collaborative project of the Institute of Public Health, Bengaluru, and JSS Medical College, Mysuru, Karnataka.

All PwSCD of tribal communities aged more than two years with a confirmed diagnosis via high-performance liquid chromatography (HPLC) and enrolled in the registry will be invited to participate. Inclusion will be contingent upon the willingness to give informed consent. Primary caregivers will be the respondents for children below 12 years of age. For the participants aged 12–18, both verbal assent from the PwSCD and written informed consent from their caregiver will be obtained. Individuals will be excluded if they are antenatal or lactating (up to six months postpartum), individuals with other forms of hemoglobinopathies, critically ill patients, those advised bed rest or hospitalised, or those with diagnosed mental health conditions requiring specialised care due to additional complexity.

This phase will begin with capacity-building sessions and a baseline assessment of acceptability, feasibility, and health-related quality of life (HRQoL) in the first month. Training will be delivered in small groups wherever feasible. If group meetings are not possible due to logistical or individual constraints, one-on-one sessions will be offered.

Participants will be engaged for 12 months following the training, including a one-month capacity-building training. Monthly telephonic follow-ups will be conducted, supplemented by in-person home visits every month. During these interactions, participants will be assessed using structured tools to assess the package’s acceptability and feasibility. Feedback on implementation challenges and suggestions for modifications will be documented to enable iterative refinements of the intervention strategy. This adaptive approach is designed to enhance real-world applicability, participants' engagement, and long-term sustainability of the self-management intervention.

***Data collection tools and analysis plan***

**Quantitative data:**

1. *Feasibility and Acceptability*-The Acceptability of the Intervention Measure (AIM) and the Feasibility Intervention Measure (FIM) will be used to assess the acceptability and feasibility of the self-management package. AIM focuses on assessing stakeholders' emotional and cognitive responses, examining how appealing, likeable, and suitable the interventions are for them. FIM includes questions designed to determine how practical and implementable interventions are in real-world settings. It assesses participants' perceptions of their ability to execute the intervention, considering factors such as effort, accessibility, and the resources required for effective implementation ^27^. Both tools are concise, user-friendly, and well-suited for low-resource community settings. Tools will be used after piloting to increase relevance by incorporating a few open-ended questions to capture specific suggestions on how the intervention could be made more acceptable or feasible within their context.
  At the end of the 12-month implementation phase, additional qualitative data will be collected to explore participants' overall experiences with the self-management package. These insights will inform further refinement of the intervention. Additionally, intervention compliance with the self-management package will be measured.
Analysis plan: Each measure will be summarised using the mean and standard deviation scores to capture participants' perceptions of acceptability and feasibility. Since there is no established cutoff score for AIM and FIM, a higher score that improves acceptability and feasibility will suggest increased acceptability and feasibility of the intervention. To align with the study objective, achieving a threshold where at least an 80% score will be considered a good indicator of success. Analysis of additional qualitative data is mentioned in the section on qualitative data.
2. *Health-related quality of life* **-**Although it is not the primary objective, a preliminary evaluation of effectiveness will be done using a standardised checklist called Short-Form 36 (SF-36). There are 36 questions in the checklist, which are divided into eight domains: general health, activity limitations, problems related to physical health, pain, emotional health problems, and social activities. This tool is validated using it in diverse populations, showing the highest internal consistency. A recent study indicates the cultural acceptability of the SF-36 tool ^28^. SF-36 guidelines will be followed for scoring ^29^. This checklist will be administered at the baseline, midterm, and final stages of implementation
Analysis plan: Information on basic demography and QoL data will be collected and compiled in an Excel sheet for analysis in JAMOVI. Participants' scores will be generated according to each domain given in the SF-36. As we track the QoL baseline at the start, mid-term, and end lines, data from the beginning of the phase will be analysed using ANOVA to examine the trends.

**Qualitative data:**

A guide for semi-structured interviews and quantitative tools will be prepared to investigate participants' experiences. Observations during home visits will be noted to explore the feasibility of a self-management package. Participants who dropped out of the study will also be invited to share their insights. Additionally, those who have demonstrated good compliance will be chosen for in-depth interviews (IDIs) and FGDs.

Analysis Plan: Qualitative data will be coded, and thematic analysis will be conducted using Qual-coder.

Both quantitative and qualitative data will be triangulated to analyse comprehensively. At the end of this phase, we expect to have a finalised, acceptable, and feasible self-management package that can be implemented further at a larger level.

The overview of both phases is shown in Table 1.

**Table 1.** Methods that will be used in both phases.

Phase	Method	Purpose	Sampling technique	Participants	Tools	Analysis Plan
Phase I	Literature review	To learn about self- management practices and components	Purposive and convenient	PwSCD/caregivers suggested by team members, Subject Experts Key persons from NGOs	PubMed, Google Scholar, Scopus, and Web of Science using the appropriate search strategy. Websites of health departments of other countries and grey literature will be reviewed. Data will be managed using Excel sheets and Zotero.	Relevant articles will be selected based on a review of the abstract.
	Qualitative method	Experts/key persons: To understand what kind of self-management practices should be addressed from an Indian perspective. PwSCD: To learn their perspective while adopting self-management practices and maintaining adherence			In-depth Interview and focus group discussion Guide	The thematic analysis will be done using the software Qual- Coder.
Phase II	Quantitative	To assess and improve the self-management package's acceptability and feasibility. To measure of QoL		All PwSCD meet the inclusion criteria of the study area	Modified Feasibility Intervention Measure Modified Acceptability Intervention Measure SF36-QoL checklist	
	Qualitative	To know barriers and facilitating factors while practising a self- management package. To know PwSCD/caregivers' experiences while using self- management.			1. In-depth interview guide 2. Semi-structured checklist	Qualitative information will be analysed using thematic analysis with the software Qual Coder.

### Ethics and dissemination

The full proposed study protocol has ethical approval from the Institutional Committee of JSS Medical College, Mysuru, Karnataka. The Institute of Public Health, Bengaluru, also approved for Phase I of the proposed study. Informed consent from participants will be obtained separately for each phase. All data, including recordings and notes, will be securely stored in an encrypted system, with access restricted to the study team. Identities will be coded, and personally identifiable information will be excluded. Anonymised data may be shared with collaborators. Research data will be securely archived for 5 years before deletion as per IEC guidelines.

The findings will be disseminated through International and National Conferences, peer-reviewed publications, Adivasi (tribal) Sangha meetings, policy briefs, and digital and local print media.

## Discussion

Self-management is a cornerstone of comprehensive care for chronic diseases, including Sickle cell disease, particularly in low- and middle-income countries (LMICs) like India, where healthcare access, quality, and continuity of care are often lacking, especially in rural and tribal settings.

In such a context, where SCD symptoms can be managed by enhancing decision-making skills of individuals with knowledge and tools for effective self-management, it is essential to reduce disease burden, improve autonomy, and mitigate direct and indirect health-related costs. Existing evidence demonstrates the effectiveness of self-management interventions in non-communicable diseases (NCDs), showing improvements in health literacy, self-efficacy, and hospitalisation reductions ^30– 32^. Several studies have also found the effectiveness of Self-Management in SCD care, which promotes self-efficacy, learning, and adherence to medicine ^15, 19, 33^. However, most interventions have been developed and tested in high-income countries. They may not be directly translatable to resource-constrained tribal populations due to substantial socio-cultural differences.

The key strength of the study lies in its participatory, multi-stakeholder approach to intervention development. By integrating perspectives from PwSCD, caregivers, local healthcare providers, and national SCD experts, the study ensures that the package is grounded in real-world settings. By incorporating qualitative inputs from PwSCD/caregivers of diverse age/occupation groups and health care providers, the study will consider locally relevant barriers to adhering to self-management practice, including financial constraints, ease of use, health literacy gaps, and limited health care access.

The proposed study has certain limitations. First, the sample size can be insufficient to detect significant changes in clinical outcomes such as pain and fever episodes. However, the primary objective is to assess acceptability, feasibility, and preliminary effectiveness, which will inform the design of future largest-scale trials. Second, participants' adherence to the intervention component may vary, particularly regarding resources. Limited social and economic priorities influence health behaviour, so this study will incorporate flexible, patient-centric methods to comply with them.

In conclusion, this protocol represents an important step towards addressing a critical gap in SCD management in tribal populations in India. By focusing on community-based, context-specific self-management strategies, the study can strengthen individual health agency and improve quality of life. The outcome will contribute to the evidence base necessary for future scale-up and integration of self-management strategies into the health care system. This study can pave the way to explore further community-based interventions.

### Patient and Public Involvement

Patients and the public were not involved in developing this study protocol. However, patients' and their caregivers' perspectives and inputs will be included in the design phase. In the next phase, they will be the key participants in capacity-building training, testing and finalising the package.

## Ethics approval letter number

**Institutional Ethics Committee of**

1. JSS Medical College, Mysore, Karnataka, India
      Letter reference number: **JSS/ MC/ PG/PHD2/2024-25**
2. Institute of Public Health, Bengaluru, Karnataka, India- Study ID: **IEC/FR/07/2024**
3. Institute of Public Health, Bengaluru, Karnataka, India- Extension of duration letter
      Study ID: **07/2024/ER**

The study started from 10/10/ 2024 after the Institutional Ethics Committee approved it.

## Data Availability

Since this is a study protocol, data are not associated with this protocol paper.
