# Supplementary material for: Equipping Persons with Sickle Cell Disease, Transforming Care: A Protocol Paper on Feasibility and Acceptability of Self-Management Package in the Tribal Communities of Southern India
Source: Wellcome Open Res. 2025 Oct 27;10:607. [Version 1] doi: 10.12688/wellcomeopenres.24369.1 (PMC12800602; doi:10.12688/wellcomeopenres.24369.1)
Supplement: Peer review history

# Reviewer report: Reviewer response for version 1

**DOI:** 10.21956/wellcomeopenres.26870.r142141

**Published:** 2026-01-13

**Authors:** Shehu Shagari Awandu

**Affiliations:** 1 Jaramogi Oginga Odinga University of Science and Technology, Bondo, Kenya

Thanks of giving me the opportunity to review the manuscript where the authors aim to develop a self-management package for tribal communities living with sickle cell disease in India.

 The methods can be improved for example the instances where few home visits are mentioned, it should be more specific and a number given or an estimate provided. 

 The authors should also work on minor typos and grammatical errors in the document for example using variance instead of variant. Persons and persons etc

 Secondly, treatment should not enhance the SCD condition but should reduce it or diminish it.

 The use of purposive and convenient sampling techniques that is dependent on rapport with PwSCD and caregivers is vague. I suggest the authors state a threshold or an estimate number of blocks they will select ? For example if they only find 1 block, will this be adequate and sufficient? 

 On FGDs, how many will be conducted and how many participants will be selected. They should provide this. Can the authors also address the issue of saturation?

 In the figure the implementation phase is 11 months while in the write up its 11 months. The authors should be consistent.

 The authors assert that they will invite all PwSCD of tribal communities aged more than 2 years with a confirmed diagnosis with HPLC and enrolled in the registry. In my view,  this indicates that with more enquiries, they can have the numbers and estimates of persons from previous years. This information is lacking and can strengthen the manuscript.

 On the qualitative data, the guide that will be prepared for semi-structured interviews and quantitative tools should be provided even as appendix.

Is the study design appropriate for the research question?

Partly

Is the rationale for, and objectives of, the study clearly described?

Yes

Are sufficient details of the methods provided to allow replication by others?

Partly

Are the datasets clearly presented in a useable and accessible format?

Not applicable

Reviewer Expertise:

Epidemiology

I confirm that I have read this submission and believe that I have an appropriate level of expertise to confirm that it is of an acceptable scientific standard, however I have significant reservations, as outlined above.

# Reviewer report: Reviewer response for version 1

**DOI:** 10.21956/wellcomeopenres.26870.r142134

**Published:** 2026-01-09

**Authors:** Michael Aondoaseer

**Affiliations:** 1 Benue State University Teaching Hospital Makurdi, Makurdi, Nigeria

The authors' intention is not clear: whether this is a protocol for a study to be carried out or if they are proposing.  The abstract is clear and conveys an intention to conduct a study.

 The background highlights the problem, but the objectives are not SMART and do not align with the topic. there is a need for more clarity and specificity on what authors intend to do.

 The methodology needs to be reviewed to align with the topic: Equipping persons with sickle cell disease.

 If you are designing a tool, what are the theoretical and conceptual frameworks?  

 The discussion section does not apply in this context because the tools have not even been designed. In designing a protocol for an intended study, the segments should include an introduction, a literature review, a methodology, a data collection plan, an ethical approval plan, and appendices

Is the study design appropriate for the research question?

Partly

Is the rationale for, and objectives of, the study clearly described?

Partly

Are sufficient details of the methods provided to allow replication by others?

No

Are the datasets clearly presented in a useable and accessible format?

No

Reviewer Expertise:

Neonatology, sickle cell disease, newborn screening, infectious diseases, infant nutrition

I confirm that I have read this submission and believe that I have an appropriate level of expertise to confirm that it is of an acceptable scientific standard, however I have significant reservations, as outlined above.

# Reviewer report: Reviewer response for version 1

**DOI:** 10.21956/wellcomeopenres.26870.r137568

**Published:** 2025-11-12

**Authors:** Kristin Haglund

**Affiliations:** 1 Marquette University, Milwaukee, Wisconsin, USA

This paper describes a well-planned study to create a needed and important intervention.  Appropriately, and importantly, people living with SCD are included in the development of the intervention.

 My comments are regarding content in the abstract, background and significance.  There are some instances that could be clarified. Increased clarity will help your reader understand better what the intent of the study is and also help the reader grasp how important it is.  The revisions I am suggesting are minor. 

 Abstract & repeated in Background

 1. the two phrases of the following sentence do not work together. It reads that self-management will work because the tribe has challenges.  But self-management works for everyone not just because of this tribe’s unique challenges – self-management works. I would suggest two sentences

 Current - “Considering the unique challenges of the tribal community, implementing self-management complementary to the prescribed clinical management for sickle cell disease care can lead to greater autonomy, improved health outcomes, and, ultimately, quality of life.”

 Suggest: Considering the unique challenges of the tribal community, will help to develop a culturally-tailored intervention that is relevant and acceptable to the people it is in intended for. Implementing self-management complementary to the prescribed clinical management for sickle cell disease care can lead to greater autonomy, improved health outcomes, and, ultimately, quality of life.

 2.

 “In response to the SCD’s burden, the Government of India launched the National Sickle Cell Anaemia Elimination Mission (NSCAEM) to eliminate Sickle Cell Anaemia before 2047. Although the initiative primarily focuses on tribal communities conducting screening, management and counselling ^10^. A significant gap exists between the tribal beneficiaries and health service provision. This is often attributed to geographical isolation, cultural differences, and inadequate healthcare infrastructure ^11, 12^.”

 When reading the above paragraph, I was actually shocked to imagine an effort to eliminate a genetic disease.  Presumably, the only way to eliminate a genetic trait would be to prevent people who carry the gene for sickle cell to not have children.  Which is not possible.  To talk about eliminating a genetic condition from tribal communities sounds draconian.  Also as you can see, pulls a reader off the topic of the paper.

 I don’t view self-management as a means to eliminate the disease from a population and probably the authors do not as well.  In fact, the intervention appears to be about preventing morbidity and mortality among people who are living with SCD.  This could be a useful thing to state right up front.

 I would suggest getting clearer about what gap the authors are referring to. As a reader, I don’t understand the gap that is referred to in the highlighted sentence.  Is the gap around the increased morbidity and mortality among people who have limited access to healthcare, and perhaps limited knowledge about their condition.  (I can see how teaching people about SCD to understand how it is inherited, could help people make their own choices about having children and in that way be somewhat preventative.  But that seems like a tangent to the planned intervention. So again clarity about the gap that is being filled and the purpose of the intervention is important so your reader understands what is happening).

  To me, it makes sense that some tribes have higher prevalence of sickle cell as the trait can be more concentrated in populations that tend to pair-bond and reproduce within their community.  I don’t know that I would call a genetic trait a disparity.  Access to healthcare is a disparity.  The disparity in mortality from SCD among nations and regions within nations – definitely a disparity.  

 3. As a researcher who has conducted several community-based self-management interventions in the community, I think this sentence in the background could be clarified.

 “However, most studies are conducted in the international context, predominantly in hospital settings, and very few are in a community context.” –

 I agree with the authors that self-management studies are international.  And disagree that they are mostly conducted in hospital settings. As a reader, “in hospital settings” connotes inpatient care.  In a recent systematic review of 32 self-management interventions for people with SCD, none were conducted in hospitals, settings included outpatient, home, web-based and community. See, Poku, B. A., Atkin, K. M., & Kirk, S. (2023). Self‐management interventions for children and young people with sickle cell disease: A systematic review. *Health Expectations*, *26*(2), 579-612.

 I do agree with the authors that specific context is necessary to develop a relevant intervention so overall, the content in this paragraph supports that need for their study. The community setting fits with other interventions as an appropriate place to develop this intervention. 

 Discussion –

 The content in the discussion is very strong.  Pulling wording from the discussion, or making the intro sections consistent with discussion, would clarify my questions in the intro sections.

Is the study design appropriate for the research question?

Yes

Is the rationale for, and objectives of, the study clearly described?

Partly

Are sufficient details of the methods provided to allow replication by others?

Yes

Are the datasets clearly presented in a useable and accessible format?

Not applicable

Reviewer Expertise:

Community-engaged research regarding self-management of sickel cell disease.

I confirm that I have read this submission and believe that I have an appropriate level of expertise to confirm that it is of an acceptable scientific standard.
